# Noninvasive Biomarkers for Cardiac Allograft Rejection Monitoring: Advances, Challenges, and Future Directions

**DOI:** 10.3390/jcm15030986

**Published:** 2026-01-26

**Authors:** Yijie Luo, Junlin Lai, Chenghao Li, Guohua Wang

**Affiliations:** 1 Department of Cardiovascular Surgery, Union Hospital, Tongji Medical College, Huazhong University of Science and Technology, Wuhan 430022, China; luoyijie9909@163.com (Y.L.);; 2 Department of Cardiovascular Surgery, Zhongnan Hospital of Wuhan University, Wuhan 430071, China

**Keywords:** allograft rejection, biomarker integration, cardiac transplantation, donor-derived cell-free DNA, gene expression profiling, microRNA, liquid biopsy, non-invasive biomarkers

## Abstract

Cardiac transplantation remains an important therapy for end-stage heart failure, although allograft rejection continues to pose significant clinical challenges. This review evaluates both established and emerging blood-based biomarkers for noninvasive monitoring of rejection in heart transplant recipients. Donor-derived cell-free DNA (ddcfDNA) and gene expression profiling (GEP) represent well-validated, commercially available molecular tools that demonstrate strong discriminative capacity for acute rejection episodes. Additionally, microRNAs (miRs) and extracellular vesicles (EVs) show considerable potential as novel biomarkers, although further validation is required. In contrast, conventional biomarkers such as B-type natriuretic peptide (BNP), cardiac troponins, and creatine kinase-MB (CK-MB) offer limited specificity in the context of rejection. This review synthesizes current evidence on the clinical utility, methodological challenges, and integration strategies of these biomarkers, highlighting a shift toward molecular-based approaches for improving post-transplant surveillance and patient outcomes.

## 1. Review Methodology

This narrative review synthesizes current evidence on noninvasive biomarkers for cardiac allograft rejection. We conducted a comprehensive literature search in PubMed, focusing primarily on publications from the past decade to capture the most recent advances, using search terms including “cardiac transplant rejection,” “donor-derived cell-free DNA,” “gene expression profiling,” “microRNA,” “extracellular vesicles,” “BNP,” “troponin,” and “noninvasive biomarker.” Articles were selected based on relevance to clinical utility, diagnostic performance, and mechanistic insights. Priority was given to clinical studies, randomized trials, meta-analyses, and recent consensus statements from ISHLT and ESOT. Given the rapid evolution of this field, this review aims to provide a critical, clinically oriented synthesis rather than a systematic appraisal.

## 2. Background

Cardiac transplantation remains an important therapeutic option for end-stage heart disease, with its clinical application continuously growing alongside advancements in immunosuppressive protocols. According to data from the International Society for Heart and Lung Transplantation (ISHLT), global annual cardiac transplant volume has exceeded 5500 cases [1]. However, acute rejection (AR) persists as a major determinant of prognosis. AR encompasses several pathological processes, primarily acute cellular rejection (ACR), mediated by T lymphocytes, and antibody-mediated rejection (AMR), driven by donor-specific antibodies, along with mixed forms featuring components of both. The 1-year post-transplant incidence of AR has been reported in various eras and registries; contemporary data indicate an incidence of approximately 12.6% between hospital discharge and the first year post-transplant [2]. Endomyocardial biopsy (EMB) currently serves as the pathological gold standard for diagnosing ACR [3]. ISHLT guidelines recommend surveillance EMB at intervals during the first 3 to 12 postoperative months [3]. Nevertheless, EMB as an invasive procedure is constrained by procedure-related adverse events, patient discomfort, low interobserver agreement in rejection grading, and limited sensitivity for detecting AMR [4,5].

Blood biomarkers could reduce patient discomfort, eliminate operator-dependent bias, and enable long-distance monitoring. Novel Molecular Biomarkers, including microRNAs (miRs), extracellular vesicles (EVs), gene expression profiling (GEP), and donor-derived cell-free DNA (ddcfDNA), are expanding diagnostic capabilities. This review summarizes recent advances in these biomarkers.

## 3. Novel Biomarkers

### 3.1. ddcfDNA

#### 3.1.1. Introduction and Biological Basis

Donor-derived cell-free DNA (ddcfDNA) is a novel liquid biopsy technology that quantifies graft injury by detecting donor-specific genetic markers (e.g., single nucleotide polymorphisms [SNPs], HLA mismatches) in peripheral blood. Its biological origins primarily involve the release of genetic material from donor heart cells (e.g., cardiomyocytes, endothelial cells) upon cell death. While processes such as cardiomyocyte necrosis/apoptosis induced by activated cytotoxic T cells in ACR and microvascular endothelial injury caused by complement activation in AMR are key drivers of this release, a variety of other conditions—including infection, heart failure, ischemia–reperfusion injury—can also induce graft injury and consequently elevate ddcfDNA levels. The percentage of ddcfDNA (%ddcfDNA) describes the amount of ddcfDNA compared with the total cfDNA found in the blood [6]. Total cfDNA is a mixture derived from both the donor organ (ddcfDNA) and the recipient's own cells (recipient-derived cfDNA). The latter originates predominantly from the turnover of hematopoietic cells, such as lymphocytes and myeloid cells within the recipient's body [7].

#### 3.1.2. Diagnostic Performance and Key Studies

As a biomarker of transplant injury, ddcfDNA has demonstrated robust diagnostic reliability, particularly a high negative predictive value (NPV) for ruling out rejection, across multiple validation studies [8,9,10,11,12]. However, the statistical basis of NPV is inherently influenced by disease prevalence; in a surveilled population with a low event rate (e.g., <5%), even a test with modest sensitivity can yield a high NPV, as seen in several ddcfDNA studies [10,11,12]. This underscores its effectiveness for ‘ruling out’ rejection in stable patients but also highlights the concurrent challenge of achieving a high positive predictive value (PPV) in the same low-prevalence setting.

Comparison of key studies reveals evolving methodologies and performance. Early work established the concordance of ddcfDNA with biopsy for significant rejection (grade ≥ 2R/3A) [8,9]. Subsequent larger cohort studies using commercial platforms (AlloSure^®^, Prospera™) consistently reported high NPVs (97–99%) but modest PPVs (9–25%) and variable sensitivities (44–80%) at different thresholds [11,12]. A landmark multicenter study (GRAfT) further validated utility, reporting significant %ddcfDNA elevation during AR episodes (median 0.38% vs. 0.03%, *p* < 0.001) with an AUC of 0.92 at a 0.25% threshold, and notably, that AMR showed 5-fold higher levels and a superior AUC (0.95) compared to ACR [6]. This highlights pathophysiological differences between rejection types that ddcfDNA may capture.

#### 3.1.3. Methodological Advances and Limitations

A key limitation of %ddcfDNA is its nature as a ratio metric, where variations in both donor- and recipient-derived cfDNA can confound interpretation (such as sepsis, malignancies, myocardial ischemia, procedural injury, infection and hemorrhage) [13,14,15,16]. To address this, recent studies have evaluated absolute quantification of ddcfDNA. Kim et al. found that using an absolute concentration threshold (≥13 copies/mL, AUC = 0.88) demonstrated superior performance compared to a %ddcfDNA threshold [12]. This approach was extended using a two-threshold algorithm (2TA)—first pioneered by Halloran's team in kidney transplantation [17]—to the field of heart transplantation by integrating both parameters, which showed improved sensitivity (86.5%) and specificity (83.6%) [18]. The work of Böhmer et al. provides critical clinical examples supporting absolute quantification, demonstrating how isolated rises in %ddcfDNA due to changes in recipient-derived cfDNA (e.g., from hemorrhage) can be misleading, whereas stable absolute ddcfDNA concentrations indicate an absence of true rejection-related injury [16]. In a larger cohort, an absolute concentration threshold outperformed %ddcfDNA for detecting symptomatic rejection (AUC 0.87 vs. 0.75) [19].

#### 3.1.4. Clinical Challenges and Standardization

Key clinical challenges remain. First, ddcfDNA interpretation is confounded by non-rejection graft injury in the first 14 post-transplant days [5]; current ISHLT guidelines note this limitation without endorsement, while ESOT emphasizes differentiated evaluation [3,20]. Second, assay cost and accessibility vary, with next-generation sequencing (NGS) being costly and slower, while droplet digital PCR (ddPCR) offers a lower-cost alternative with absolute quantification [5,19]. Third, significant biological variability exists across populations (e.g., adults vs. children) and is influenced by confounders like BMI and sex, necessitating consideration of individualized thresholds [5,19]. Furthermore, ddcfDNA cannot differentiate ACR from AMR, and conditions like infection can cause false positives [5,21]. A major barrier to broader adoption is the lack of assay standardization across platforms and laboratories, a challenge acknowledged by recent consensus statements [20].

#### 3.1.5. Decentralized ddcfDNA Platforms

Decentralized or point-of-care platforms (e.g., AlloSeq^®^) represent an emerging logistical model for ddcfDNA testing. In kidney and liver transplantation, such platforms have shown potential to maintain diagnostic accuracy while improving test turnaround and accessibility [22,23]. To date, heart transplant-specific clinical validation data are not yet available. If successfully validated for cardiac allograft monitoring, decentralized testing could facilitate faster results in acute settings and broaden access to routine surveillance. Translation into heart transplantation practice will require dedicated studies to confirm performance and define appropriate clinical integration pathways.

### 3.2. Gene Expression Profiling (GEP)

Deng et al. developed an 11-gene classifier for acute cardiac allograft rejection using a custom microarray of 7370 genes, which formed the basis of the AlloMap^®^ test (CareDx, Inc., Brisbane, CA, USA). The test quantifies expression of these genes from peripheral blood to generate a score from 0 to 40, with higher scores indicating increased rejection risk. In validation on 63 independent samples, the model showed 84% concordance (95% CI: 66–94%) for detecting grade ≥3A rejection, but could not identify mild rejection (ISHLT 1A/1B) [24].

Pham et al. conducted a landmark randomized controlled trial (RCT) evaluating AlloMap^®^ clinical utility in 602 cardiac transplant recipients who were at least 6 months post-transplant [25]. Consequently, the findings are not generalizable to the early post-transplant period (<6 months). Participants were randomized 1:1 to either GEP-guided monitoring (AlloMap^®^ testing with selective biopsies based on score thresholds and clinical parameters) or standard EMB surveillance. At a median follow-up of 19 months, the GEP-guided strategy demonstrated non-inferiority to routine EMB surveillance, evidenced by a comparable 2-year cumulative incidence of the composite primary endpoint—defined as death, graft loss, or rejection with hemodynamic compromise—of 14.5% in the GEP group versus 15.3% in the EMB group, alongside an all-cause mortality hazard ratio (HR) of 1.04. This paradigm shift to GEP-guided management significantly reduced biopsy frequency (median 3 vs. 15 procedures/patient, *p* < 0.001) while maintaining equivalent clinical outcomes [25]. The integrated approach of serial AlloMap^®^ scoring and clinical assessment enhanced patient tolerance by minimizing invasive procedures without compromising safety.

Emerging strategies propose synergistic combination of ddcfDNA and GEP to leverage their complementary diagnostic strengths [24,26]. Rodgers et al. pioneered dual-testing evaluation, observing significantly reduced specificity (47%) when combining both biomarkers [26]. Conversely, Khush et al. demonstrated that combinatorial analysis could enhance diagnostic precision [27]. They reported that a dual-positive (GEP+/ddcfDNA+) status for ACR, while exhibiting reduced sensitivity (32.3%) compared to either test alone (59.0% for GEP and 44.7% for ddcfDNA), achieved superior specificity (91.7% vs. 56.8% and 84.6%, respectively) and a higher positive likelihood ratio (3.90 vs. 1.37 and 2.91). Furthermore, dual-negative results were highly reassuring, predicting a less than 1% probability of subsequent biopsy-proven ACR [27]. These contradictory findings likely stem from methodological variances in assay platforms and data interpretation algorithms. The clinical utility of integrating complementary biomarker platforms to enhance diagnostic accuracy requires rigorous investigation.

### 3.3. MicroRNA

#### 3.3.1. Potential and Early Promising Findings

MicroRNAs (miRs) are a group of highly conserved small non-coding RNAs (sncRNAs) that negatively regulate protein synthesis by binding to the 3′ untranslated region of mRNAs [28]. Some miRs are expressed in a tissue-specific manner [29] and can be released into bodily fluids through various mechanisms such as extracellular vesicles, necrosis, and apoptosis [30,31]. Furthermore, miRs exhibit high biological stability, with no significant degradation even after more than 15 years of ultra-low temperature storage [32]. These characteristics make miRs promising non-invasive biomarkers for detecting transplant rejection.

Multiple exploratory studies have reported associations between specific miRs and cardiac allograft rejection. For instance, differential expression of miRs like miR-155 and miR-182 was identified in murine transplant models [33]. In human studies, panels including miR-10a, miR-31, miR-92a, and miR-155 showed high discriminatory capacity (AUCs 0.93–0.998) [34]. Other candidates like miR-29, miR-223, miR-181a-5p, miR-144-3p and miR-652-3p have also been correlated with rejection severity [35,36,37,38,39,40,41,42,43]. Mechanistic studies, particularly on miR-155, have linked it to promoting immune rejection by targeting pathways involved in lymphocyte proliferation and Th17 cell differentiation [44,45,46,47].

#### 3.3.2. Critical Appraisal of Conflicting Evidence

The promising diagnostic performance reported in these exploratory studies stands in stark contrast to the findings of a rigorous prospective multicenter investigation by Coutance et al. [48]. This study systematically evaluated a panel of nine candidate miRNAs (including miR-10a, miR-92a, and miR-155) in 150 heart transplant recipients and found that none demonstrated sufficient discriminative power to reliably distinguish rejection from non-rejection, leading the authors to conclude they lack clinical utility in their current form.

This significant discrepancy necessitates critical analysis. Key factors likely include: (1) Study Scale and Design: Many positive studies were single-center with relatively small sample sizes, increasing susceptibility to overfitting and overestimation of effect sizes, whereas the negative study was a large prospective multicenter trial. (2) Methodological Heterogeneity: A lack of standardization in sample collection, RNA extraction, normalization strategies, and quantification platforms across studies hampers comparability. (3) Cohort Differences: Variations in rejection prevalence, severity, time post-transplant, immunosuppression, and confounding conditions (e.g., infection) can profoundly influence miRNA expression patterns. (4) Publication Bias: A tendency for positive findings to be published more readily than negative ones. The Coutance et al. study underscores the critical gap between discovery-phase research and clinically validated applications, highlighting the need for large-scale, prospective validation under standardized conditions.

### 3.4. Extracellular Vesicles

Extracellular vesicles (EVs) are a heterogeneous population of membrane-bound structures derived from cells, present in biological fluids, and involved in various physiological and pathological processes. They are classified into exosomes (originating from the endosomal system) and microvesicles (shed from the plasma membrane) [49]. EVs are recognized as a mechanism of intercellular communication, enabling the exchange of proteins, lipids, and genetic material between cells [50]. In immunity, EVs have been shown to contribute to antigen presentation and immune activation [51,52].

They show potential as biomarkers by carrying donor antigens, immunomodulatory molecules, and nucleic acids that reflect the graft's immune status. Studies have observed allogeneic EVs promoting B cell activation [53], identified differentially expressed exosomal proteins in rejection [54], and found elevations of cardiac autoantigens in chronic rejection [55,56]. Surface marker analysis, such as decreased exosomal MHC class I during rejection, has shown diagnostic potential (AUC 0.934) [57]. These surface interactions facilitate antigen presentation and immune cell communication, as shown by studies detailing how EVs bind to recipient cells via specific surface proteins [58,59]. Furthermore, the cargo of EVs can reflect specific immune cell activity. Profiling of circulating T cell-derived EVs has revealed significant upregulation of immunoregulatory markers and associated microRNAs during rejection, offering a novel avenue for immune cell-specific monitoring [60]. Similarly, donor heart-derived dendritic cells in mouse models have been shown to activate recipient T cells via secretion of EVs carrying tetraspanins CD9 and CD63 [61], further elucidating a specific cellular mechanism by which graft-derived EVs can drive alloimmunity. A recent study using an AI model to analyze EV surface antigen "fingerprints" achieved an accuracy of 78.9% and an AUC of 0.832 for detecting ACR [62].

Notwithstanding the significant progress in non-invasive diagnosis of cardiac transplant rejection using EVs, which demonstrates high sensitivity, specificity, and great potential for obtaining information non-invasively, several key challenges remain in this field. Firstly, techniques for the isolation, purification, and standardized detection of EVs lack uniformity, and differences in platforms and methodologies across studies hinder direct comparison and clinical translation. Secondly, most existing studies are based on small cohorts, necessitating validation through larger-scale, multi-center prospective clinical trials. Future research should focus on promoting the standardization and automation of EV detection technologies to establish stable and reproducible operational protocols. Concurrently, integrating multi-omics data (e.g., proteomics, miRNAomics, surface antigen profiling) with artificial intelligence algorithms will be essential for constructing more accurate dynamic diagnostic and early-warning models. Furthermore, in-depth investigation into the biological functions of EVs and their specific roles in immune rejection mechanisms will facilitate the discovery of novel therapeutic targets and advance their development toward integrated "diagnosis-therapy" applications.

## 4. Conventional Biomarkers

### 4.1. BNP

B-type natriuretic peptide (BNP) is a hormone released by ventricular cardiomyocytes in response to stretch. While it is a cornerstone biomarker in heart failure management, its role in heart transplant recipients is complex: BNP levels are elevated compared to healthy controls [63,64,65,66,67], further increase during rejection episodes [68,69,70,71,72,73], and decrease after immunosuppressive therapy [74,75], suggesting involvement in transplant immunomodulation.

Mechanistically, proinflammatory cytokines (e.g., IL-1β, TNF-α) enhance BNP expression and secretion in cardiomyocytes [76], while exogenous BNP can modulate immune cell counts and cytokine production [77,78]. BNP also correlates with specific cytokines and shares signaling pathways (e.g., NF-κB) during high-grade rejection [79]. Combining BNP with soluble ST2 (sST2) improved rejection prediction [80].

Despite some studies reporting correlations between BNP/NT-proBNP elevation and rejection severity [69,81], its diagnostic accuracy remains suboptimal, particularly for AMR and biopsy-negative rejection [70,82,83]. Other studies found no significant association [84,85]. Due to substantial interindividual variability influenced by age, sex, renal function, and assay platforms [86], universal BNP thresholds are impractical; longitudinal monitoring of individualized trends may offer greater clinical utility.

In addition to hemodynamic and cytokine regulation, BNP expression is directly controlled by key transcription factors. Recent research has highlighted the central role of Serum Response Factor (SRF) in this process. SRF is a transcription factor whose expression increases during cardiac development, hypertrophy, and aging. In a transgenic mouse model with cardiac-specific SRF overexpression, a pronounced and sustained upregulation of left ventricular BNP protein levels was observed, coinciding with accelerated aging phenotypes such as cardiac hypertrophy, fibrosis, and mitochondrial dysfunction [87]. This finding establishes SRF as an upstream regulator of BNP transcription. In the context of cardiac transplantation, immune activation and tissue injury during rejection likely activate transcriptional pathways, including SRF signaling, directly driving BNP synthesis and release from cardiomyocytes. Consequently, monitoring BNP levels may reflect not only ventricular wall stress but also indirectly capture the activity of specific molecular pathways (e.g., SRF signaling) triggered by rejection-related stress. This provides a deeper mechanistic understanding of BNP elevation during rejection and underscores its potential value as an integrative “stress-response” biomarker.

### 4.2. CK-MB and Cardiac Troponins

Creatine kinase-MB (CK-MB) and cardiac troponins (cTn) are established biomarkers of myocardial injury. During ACR, serum CK-MB may rise [88,89,90], but its diagnostic performance is inconsistent, with some studies reporting limited sensitivity and specificity [88,91]. Consequently, the 2022 ISHLT guidelines do not recommend CK-MB for rejection monitoring (Class III) [3].

Cardiac troponins, particularly high-sensitivity assays (hs-cTn), show greater promise. Elevated troponin levels are associated with moderate-to-severe rejection and demonstrate high NPV for excluding AR [92,93,94,95,96]. For example, a troponin T (TnT) cut-off of 15 ng/L achieved 91.3% sensitivity and 88.3% specificity for severe rejection [97]. Hs-cTn assays further improve sensitivity and NPV, supporting their role as effective rule-out tests for ACR [95,98].

## 5. Discussion

The evidence synthesized in this review underscores a paradigm shift in cardiac allograft rejection surveillance, moving from invasive histological assessment toward integrated, non-invasive biomarker-driven strategies. This discussion synthesizes the clinical utility, limitations, and strategic integration of these biomarkers, with a focus on their practical application.

### 5.1. Clinical Application and Timing of Biomarker Use

The optimal use of non-invasive biomarkers is highly dependent on the post-transplant phase and the clinical question. In the early post-operative period (<1 month), nonspecific graft injury can confound ddcfDNA interpretation, limiting its utility despite its high sensitivity for subsequent rejection [5,6,20]. During this phase, the assessment for rejection still necessitates a comprehensive approach, including EMB with clinical symptoms, serological tests, and echocardiography. For stable patients beyond 6 months, Gene Expression Profiling (AlloMap^®^) has demonstrated robust clinical utility, significantly reducing biopsy frequency without compromising outcomes, and is best suited for monitoring low-risk, stable recipients to rule out subclinical ACR [25]. DdcfDNA demonstrates high sensitivity for both ACR and AMR, often preceding histological evidence, making it particularly valuable for rejection surveillance in the first year, for monitoring high-risk patients, and for investigating suspected AMR or graft dysfunction when biopsy is equivocal or high-risk [6,9,12]. Its ability to provide an early signal of injury supports its role in pre-emptive management. Conventional biomarkers like hs-cTn, with their high NPV, can serve as ancillary rule-out tools, while BNP's role is largely confined to trend monitoring due to poor specificity.

### 5.2. Integration Strategies and Combined Biomarker Approaches

Given that no single biomarker is perfect, combined approaches are emerging to enhance diagnostic precision. The complementary nature of ddcfDNA and GEP provides a logical basis for dual-marker strategies. Evidence suggests that dual-negative (ddcfDNA−/GEP−) results are highly effective for ruling out active rejection, potentially enabling a significant reduction in surveillance biopsies [27]. In contrast, a dual-positive (ddcfDNA+/GEP+) result substantially increases the specificity and probability of rejection, warranting further investigation or treatment escalation [27]. However, the diagnostic performance of such combinations can vary with the assay platforms and algorithms used, as evidenced by conflicting study results [26,27]. Future clinical protocols may leverage these combinations to create risk-stratified pathways, where biomarker results, integrated with clinical and echocardiographic data, guide the decision to forgo, perform, or treat based on biopsy.

**Figure 1:**
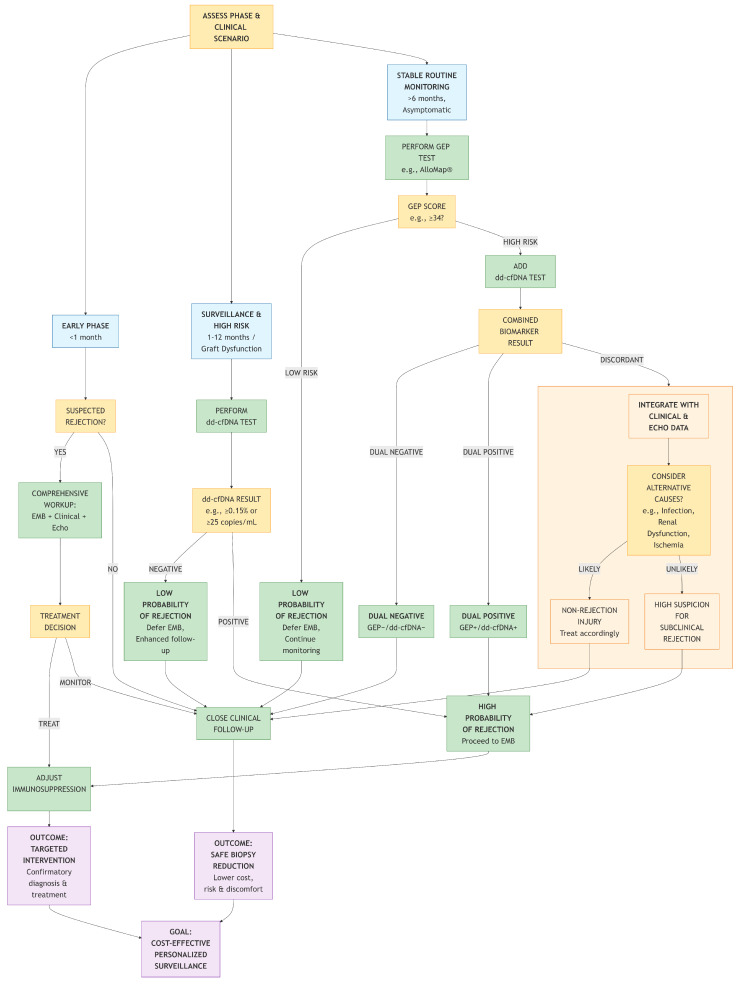
Clinical Decision Pathway for Noninvasive Biomarker-Guided Surveillance in Heart Transplant Recipients.

To synthesize the evidence and clinical guidance discussed above, we propose a practical decision pathway (Figure 1) for noninvasive biomarker-guided surveillance in heart transplant recipients.

### 5.3. Limitations and Standardization Challenges

Widespread adoption of these novel biomarkers is hampered by several challenges. A primary concern is the lack of standardization, with significant inter-assay and inter-laboratory variability in ddcfDNA reporting thresholds and GEP score interpretations [6,10,11,12]. The high cost of ddcfDNA testing and the limited reimbursement policies in many healthcare systems further restrict its accessibility. For GEP, its lower accuracy in the early post-transplant period and for detecting AMR confines its use to a specific patient subset [24,25]. Promising research biomarkers, such as microRNAs and extracellular vesicles, face even greater hurdles, including the absence of standardized isolation protocols, analytical heterogeneity, and a lack of validation in large, multicenter cohorts [48,62]. Conventional biomarkers, including BNP and cardiac troponins, despite their availability, exhibit poor specificity for rejection due to significant confounding by concomitant conditions such as infection, renal dysfunction, and ischemia [86,98,99].

### 5.4. Divergence in Current Guidelines and Paths for Evolution

The integration of non-invasive biomarkers into clinical practice is reflected in, yet also challenged by, current international guidelines, which themselves reveal areas of evolving evidence and clinical judgment. A comparative analysis of the recent ISHLT guidelines and the ESOT consensus statement highlights both convergence and notable divergences. Both bodies recognize the high negative predictive value of ddcfDNA for ruling out rejection and the utility of GEP (AlloMap^®^) in stable, late-phase patients to reduce biopsy burden. However, their recommendations differ in strength and specificity. For ddcfDNA, the 2023 ISHLT guidelines describe its performance and emerging use cautiously without a formal class recommendation, acknowledging its potential while noting limitations like early post-operative confounding [3]. In contrast, the ESOT consensus provides a structured but weak recommendation for ddcfDNA use beyond 4 weeks post-transplant, explicitly citing the lack of randomized trial data and concerns about assay standardization and variability between centralized platforms [20]. Regarding GEP, ISHLT offers a Class IIa recommendation [3], whereas ESOT assigns a strong recommendation for its use in ruling out ACR in stable patients, tempered by the pragmatic caveat of its unavailability in Europe [20]. For conventional biomarkers like high-sensitivity troponin, ISHLT notes its promising NPV [3], while ESOT assigns a weak neutral recommendation due to conflicting data [20].

These discrepancies stem from several factors: differences in the weighting of available evidence (e.g., reliance on randomized trials for GEP vs. large cohort studies for ddcfDNA), variable interpretation of test performance in low-prevalence settings, and regional considerations of test access and cost.

The trajectory of future guidelines will likely be shaped by several key developments. First, prospective data on the combined use of ddcfDNA and GEP, and their integration into clinical decision pathways, will be crucial for defining precise roles beyond solitary use. Second, the maturation of decentralized testing platforms and the generation of robust, real-world performance data outside research settings will address current concerns about standardization and accessibility. Third, evidence regarding the use of these biomarkers for dynamic risk stratification and therapy guidance—such as modulating immunosuppression in response to biomarker trends—is eagerly awaited and could transform their application from passive surveillance to active management tools. As this evidence accumulates, future guidelines are poised to move from cautious acknowledgment to more definitive, nuanced, and potentially biomarker-combination-stratified recommendations for personalized post-transplant care.

A comparative overview of the mechanisms, strengths, and limitations of these noninvasive biomarkers is presented in Table 1.

## 6. Future Directions

To overcome existing barriers and fully realize the potential of non-invasive monitoring, future efforts must focus on several key areas. First, establishing standardized pre-analytical and analytical protocols across platforms is essential for ensuring result reproducibility and comparability. Second, prospective, multicenter studies in diverse populations are urgently needed to validate integrated biomarker panels and define their cost-effectiveness. Finally, ongoing research into the biological mechanisms underpinning biomarkers like EVs and miRs will not only solidify their diagnostic roles but may also unveil novel therapeutic targets.

Building upon the current momentum in biomarker research, future refinement will focus on enhancing the precision of existing tools and developing sophisticated integration frameworks. This includes advancing beyond simple quantification of ddcfDNA by exploring its biological nuances, such as its cellular origins and fragmentomic patterns, to improve specificity for rejection subtypes and distinguish it from other causes of graft injury [6,12,16]. Furthermore, the development of integration strategies that extend past the current two-threshold algorithm (2TA) is crucial. The integration of multi-dimensional data from ddcfDNA, GEP, proteomics, and metabolomics within machine-learning frameworks shows promise for building dynamic, personalized risk models [6,12,27,62], as exemplified by recent work combining EV surface-antigen profiling with AI-assisted analysis [34,100]. Additionally, spatial transcriptomics and single-cell technologies offer new avenues to resolve immune-microenvironment heterogeneity in rejecting grafts and identify cell-type-specific therapeutic targets [101,102,103].

## 7. Conclusions

In conclusion, non-invasive biomarkers are redefining the landscape of cardiac allograft surveillance. DdcfDNA and GEP have matured into clinically validated tools that can strategically reduce reliance on invasive biopsies. Their judicious application, and potentially their combination, should be guided by the post-transplant timeline and the individual patient's risk profile. While challenges of standardization, cost, and validation remain, the trajectory is clear: the future of rejection monitoring lies in the personalized, multimodal integration of molecular signals. Through continued collaboration and rigorous validation, these innovations promise to minimize invasive procedures, optimize immunosuppression, and ultimately improve long-term graft survival.

## Figures and Tables

**Table 1:** A comparative overview of the mechanisms, strengths, and limitations of mentioned biomarkers.

Biomarker	Mechanism of Action	Key Strengths	Key Limitations
Donor-derived cell-free DNA (ddcfDNA)	Released into the bloodstream upon death (necrosis/apoptosis) of graft cells (e.g., cardiomyocytes, endothelial cells).	- High sensitivity & NPV; can detect injury early (prior to histology).	- Limited specificity: Elevation can occur from non-rejection events (e.g., infection, ischemia).
		- Detects both ACR and AMR.	- Difficult to interpret in the early post-operative period (<14 days).
		- Serves as a sensitive indicator for all-cause graft injury.	- High cost; assay standardization is ongoing.
Gene Expression Profiling (GEP)	Measures changes in gene expression in recipient's peripheral blood leukocytes in response to the graft.	- High NPV for ACR in stable patients.	- Cannot detect AMR.
		- Significantly reduces biopsy frequency, improving patient comfort.	- Cannot be used in the early post-transplant period (<2 months).
		- Commercially available and validated assay (AlloMap^®^).	- Infection can cause false-positive results (score elevation).
MicroRNAs (miRs)	Stable non-coding RNAs that regulate rejection-related pathways (immune cell activation, proliferation, myocardial injury) by targeting mRNAs.	- High stability, easily stored long-term in body fluids.	- Primarily research stage; lacks standardization and uniform signature.
		- Some miRs show very high diagnostic efficacy (AUC >0.95).	- High heterogeneity across studies; requires large-scale validation.
		- Potential to reveal rejection mechanisms and provide therapeutic targets.	- Long path from discovery to clinical translation.
Extracellular Vesicles (EVs)	Membrane-bound vesicles released by cells, carrying donor antigens, immunomodulatory molecules, and nucleic acids.	- Provides unique, cell function-related information (surface antigens, cargo).	- Technical bottlenecks: Complex, non-uniform isolation and characterization methods.
		- Potential to non-invasively monitor immune cell activation.	- Cumbersome analysis, challenging for routine clinical use.
		- A cutting-edge frontier in liquid biopsy and immune monitoring.	- Currently confined to research exploration; lacks standardized assays.
Conventional Biomarkers (BNP, hs-cTn)	BNP: Released by ventricular cardiomyocytes in response to wall stress.	- Widely available, rapid turnaround, low cost.	- Very low specificity; confounded by numerous non-rejection factors.
	hs-cTn: Structural proteins released upon myocardial cell injury.	- hs-cTn has high NPV, useful for ruling out acute rejection.	- BNP has poor diagnostic accuracy for rejection and should not be used alone.
		- Provides complementary information on hemodynamics and myocardial injury.	- Primarily useful for ancillary assessment and trend monitoring.

## Data Availability

No new data were created or analyzed in this study.

## Abbreviations

ddcfDNA	Donor-derived cell-free DNA
GEP	Gene expression profiling
miRs	MicroRNA
EVs	Extracellular vesicles
AR	Acute rejection
ACR	Acute cellular rejection
AMR	Antibody-mediated rejection
EMB	Endomyocardial biopsy
hs-cTn	High-sensitivity cardiac troponin
BNP	B-type natriuretic peptide
CK-MB	Creatine kinase-MB
PPV	Positive predictive value
NPV	Negative predictive value
